# Detection of Anomalies in Daily Activities Using Data from Smart Meters

**DOI:** 10.3390/s24020515

**Published:** 2024-01-14

**Authors:** Álvaro Hernández, Rubén Nieto, Laura de Diego-Otón, María Carmen Pérez-Rubio, José M. Villadangos-Carrizo, Daniel Pizarro, Jesús Ureña

**Affiliations:** 1 Electronics Department, University of Alcala, 28801 Alcalá de Henares, Spain; laura.diego@uah.es (L.d.D.-O.); mcarmen.perezr@uah.es (M.C.P.-R.); jm.villadangos@uah.es (J.M.V.-C.); daniel.pizarro@uah.es (D.P.); 2 Electronics Technology Department, Rey Juan Carlos University, 28933 Móstoles, Spain; ruben.nieto@urjc.es

**Keywords:** non-intrusive load monitoring (NILM), anomaly detection, predictive models, smart meters

## Abstract

The massive deployment of smart meters in most Western countries in recent decades has allowed the creation and development of a significant variety of applications, mainly related to efficient energy management. The information provided about energy consumption has also been dedicated to the areas of social work and health. In this context, smart meters are considered single-point non-intrusive sensors that might be used to monitor the behaviour and activity patterns of people living in a household. This work describes the design of a short-term behavioural alarm generator based on the processing of energy consumption data coming from a commercial smart meter. The device captured data from a household for a period of six months, thus providing the consumption disaggregated per appliance at an interval of one hour. These data were used to train different intelligent systems, capable of estimating the predicted consumption for the next one-hour interval. Four different approaches have been considered and compared when designing the prediction system: a recurrent neural network, a convolutional neural network, a random forest, and a decision tree. By statistically analysing these predictions and the actual final energy consumption measurements, anomalies can be detected in the undertaking of three different daily activities: sleeping, breakfast, and lunch. The recurrent neural network achieves an F1-score of 0.8 in the detection of these anomalies for the household under analysis, outperforming other approaches. The proposal might be applied to the generation of a short-term alarm, which can be involved in future deployments and developments in the field of ambient assisted living.

## 1. Introduction

In recent years, the aging of population has become a key aspect of the definition of future policies and sustainable healthcare systems in Europe, a continent where people aged over 50 years old are actually 37% of the population. Moreover, EC (European Commission) population estimates predict that the number of people over 60 will increase by about two million -per year over the coming decades, and by 2060, this group is expected to represent around 30% of the total population [1]. The impact of this aging population on public finances and on future public health services is assumed to be huge. In this context, cognitive disorders and impairments already have high care demands, and are becoming a significant health challenge [2]. These disorders will provoke increased demand for long-term care, wherein a crucial issue is the sustainability of coming healthcare systems as well as their cost in terms of gross domestic product. 

On the other hand, the deployment of smart meters in most Western countries has boosted the proposal and development of multiple services and applications, many related to the customers’ efficient energy management or energy demand response [3,4]. These applications have spread worldwide, partly due to the current climate emergency and energy shortages. Non-intrusive load-monitoring (NILM) techniques are focused on the energy disaggregation for each electrical load [5,6]. These disaggregated consumption data can be used not only for energy management issues [7], but also for other applications [8]. One of the most significant methods deals with the social and health aspects of aging society, wherein disaggregated load consumption might be used to infer behaviour patterns [9] or to detect anomalies for alarm generation [10,11]. In some cases, smart plugs and/or smart meters may be involved in the measurement deployment, since they provide the disaggregated data per device [12]. Nevertheless, NILM techniques offer high scalability and low intrusiveness without including any additional equipment.

In general terms, NILM techniques are often based on the detection of any change in current or power signals [13], where features are extracted [14,15] to identify loads by applying different classification methods [7,16,17]. From that point, human activity can be inferred based on the usage pattern of some appliances, as they are strongly related to certain daily activities [18,19,20]. Health aspects may also be studied from electrical consumption data, such as possible inactivity, sleep disorders, or low-activity routines [21]. These systems have been proven a suitable tool for the detection of unusual activity patterns, as well as for launching alerts to caregivers or relatives [22].

One of the first works dealing with behaviour monitoring based on electrical consumption was [23], where a unique electrical signature was identified for each person’s activity, which may provide information about their health status. A Markov model was applied in [17] to identify the activity chain based on low-sampling-energy measurements. In [18], the Dempster–Shafer theory is applied to provide a daily score about the normality of a day, when comparing it with a regular behaviour or pattern. The same approach, based on the Dempster–Shafer theory, is proposed in [24] to recognize activities such as preparing breakfast, relaxing, preparing dinner, or showering. On the other hand, in [25], a classifier based on a support vector machine (SVM) and random forest allows energy to be disaggregated in three houses, which are dedicated to training a model. The resulting trained model is then used to monitor two patients with dementia, deriving information about behavioural patterns and modifications in routines. In [26], a regression approach (based on support vector regression and linear regression) is proposed to estimate the expected energy consumption, and then, by comparing with the real one, it detects possible anomalies and launches alarms. A similar application can be found in [27], where the detection of absence from the home is performed in a straightforward manner by monitoring the use of electrical appliances instead of deploying more intrusive sensors in the household. Different machine learning techniques were compared for that purpose, such as decision tables, random forests, naïve Bayes, and neural networks. Finally, in [11], four different models (Seq2Seq, Seq2Point, temporal pooling, and the UNET-NILM model) were applied to the monitoring and evaluation of daily activities, focusing the study on patterns of kettle usage, since it is a suitable representative of hand-operated appliances. 

Another interesting approach related to behaviour monitoring and possible anomaly detection is the forecasting of the energy consumption in the household. Many previous works can be found in the literature [28], often depending on the time scale of the prediction. In [29], an LSTM (long short-term memory) network is proposed and tuned for consumption forecasting in a household, as a basis for an intelligent energy management system. The same type of neural network is combined with empirical wavelet transform for the same purpose in [30]. In [31], the use of singular spectral analysis is proposed to mitigate the effect of the variability of energy consumption on the forecasting performance of parallel LSTM networks. Finally, a comparison between an LSTM, a multi-layer GRU, and a Drop-GRU network is carried out in [32], in the context of energy consumption forecasting, wherein the LSTM approach achieved better performance.

A last significant domain of application for energy prediction in general is the optimization of energy management and household efficiency by allowing scheduling and flexibility not only in the usage of electrical appliances by end users, but also in the energy generation decisions of companies. In [33], a process is proposed for detecting the occupancy of residential buildings from their smart meter data. In the same context, short-term load forecasting is developed in [34], based on several approaches, whereas machine learning methods are the most commonly used techniques [35]. Similarly, in [36], a temporal fusion transformer is proposed to forecast the energy consumption demand in a household, improving the performance given by LSTM and temporal convolutional networks.

This work proposes the detection of anomalies in the behaviour of tenants in a household based on electrical measurements coming from a smart meter installed at its entrance. The energy disaggregated per appliance is used in the proposal to assess the performance of the household’s tenants in some daily activities in the short term, such as sleeping, breakfast, or lunch. For that purpose, four machine learning algorithms have been defined for the regression of the expected energy consumption, according to the training data from a previous period. Two approaches based on neural networks have been considered at this point: one based on convolutional networks and another on recurrent networks. For comparison’s sake, a decision tree and a random forest have also been implemented. Furthermore, a short-term alarm generator has been developed, based on statistical considerations of the aforementioned activities throughout the period under study. The performance metrics for these alarms have achieved an accuracy over 99% and an F1-score of 80%. These contributions can then be summarized as follows:

- The study of different machine learning algorithms capable of predicting the energy consumption of a household after a training period. Four algorithms are considered hereinafter: a convolutional neural network (CNN), a long short-term memory (LSTM) network, a decision tree (DT), and a random forest (RF).
- The definition of short-term alarm, which is able to launch warnings when there is a divergence in the house between the predicted energy consumption and the real measured one, during the test period after training. In particular, three alarms have been implemented related to the following daily activities: sleeping, breakfast and lunch.
- The proposal has been successfully validated with experimental data coming from a real household with four tenants. A commercial on-the-shelf smart meter has been used to acquire electrical samples during a period of six months, which proves the feasibility and readiness for implementation of the algorithms and methods described here.

The rest of the manuscript is organized as follows: Section 2 describes the proposed global architecture; Section 3 presents the energy prediction model, as well as the corresponding experimental results from a real living scenario; Section 4 details the method proposed for short-term alarm generation in daily activities; Section 5 deals with the limitations, the open challenges and the future works of the proposal; and, finally, conclusions are discussed in Section 6.

## 2. Global Architecture Overview

The proposed system is based on a smart meter, devices widely deployed in buildings and households in developed countries, which is in charge of acquiring voltage and current signals from the mains. These signals have already been analysed in the literature for NILM applications, where different alternatives have been proposed for their feature extraction. The detection of events (switching on/off of appliances connected to the mains) is likely the most common technique used to avoid continuous processing of incoming signals. Since the events are detected, only an interval of the acquired signals around the event is processed afterwards to identify the load (or appliance). The load identification can be implemented by means of different approaches, such as a Bayesian approach, principal component analysis (PCA) [10], clustering, SVM [23], or deep neural networks [15,16,37], which have become more relevant recently.

Apart from identifying loads, it is possible to deal with the detection of anomalies in usage patterns in a minimally intrusive way, in order to launch short-term alarms that may imply that the a member of the household is not following their ordinary routine in a certain daily activity, and thus might be considered to be in a risky situation to be tackled.

For that purpose, this work is based on a commercial smart meter that already provides the disaggregated energy per appliance, by accessing their company’s cloud services. It is the Wibeee Box Mono [38] that gives the appliances’ consumption per hour. As mentioned before, it has been installed in a household with four tenants (two adults and two teenagers). The energy consumption data are uploaded to the cloud by the Wibeee Box Mono; afterwards, a monitor obtains the energy consumption samples from the last days every hour, in order to forecast the actual consumption values and compare it with the real measured ones. The energy samples are not preprocessed in any way, and feature selection is not implemented before the prediction model. This one directly deals with the raw samples coming from the smart meter every hour. 

**Figure 1:**
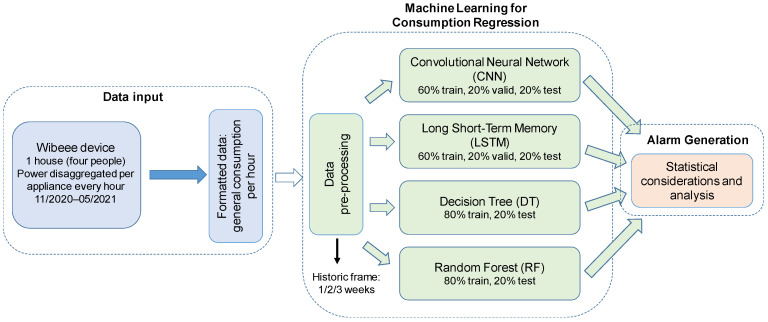
Global architecture of the proposed system.

This proposal is intended to detect non-ordinary situations wherein the household’s consumption is not following the typical daily routine in a significant way, so a short-term alarm might be generated to warn about the anomalous behaviour. This short-term alarm is implemented according to the block diagram shown in Figure 1. The proposal is based on an intelligent system, where four different approaches have been considered: a CNN, an LSTM network, a DT, and an RF. The intelligent system has been trained with the data from the Wibeee smart box installed in the house. The energy disaggregated per appliance is sampled every hour, for a period from November 2020 until May 2021. It is worth noting that these machine learning techniques were chosen due to their relatively reduced complexity and straightforward procedure, thanks to which they may be implemented on the edge (using general-purpose processors or FPGA devices), compared with other recent topologies, such as auto encoders [39]. This is a key aspect, since a last objective is indeed to integrate the whole proposal in a low-complexity smart meter [40,41].

## 3. Energy Consumption Prediction Model

**Figure 2:**
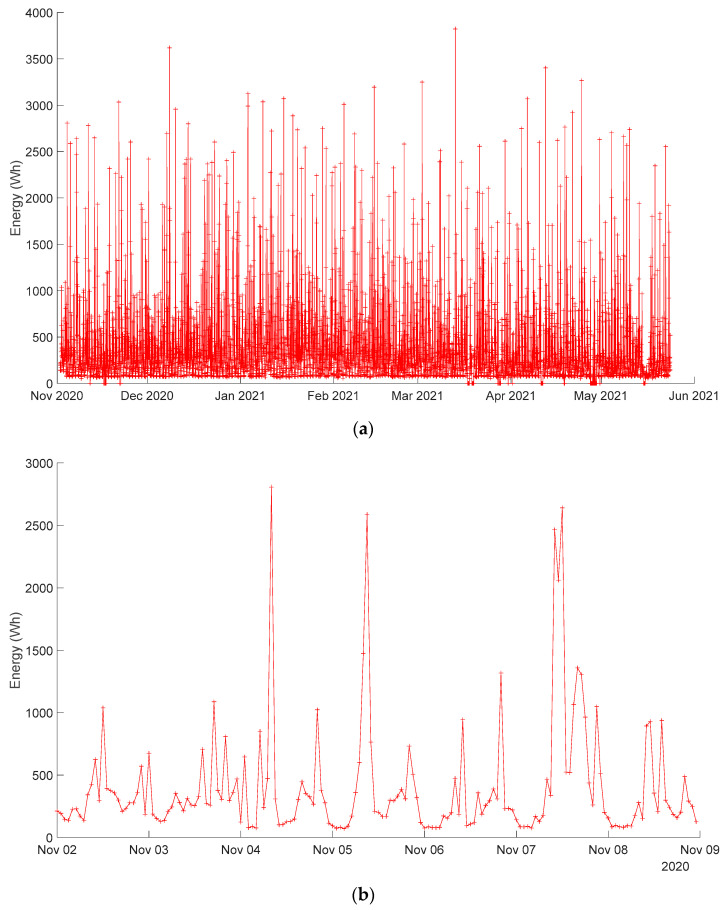
Available input data (energy consumed each hour) from the Wibeee device installed in a test house with four tenants: (**a**) the whole period under analysis; (**b**) the first week under analysis.

Figure 2 shows the input data coming from the Wibeee device, where the energy is plotted every hour. It is worth mentioning that the samples provided by the meter correspond to the energy consumed during that hour internal (units in W·h). These energy samples per hour are equal numerically to the average power, in Watts, during that hour. The objective is to train the intelligent system to predict the global consumption in the next hour interval (a regression approach), according to an input window with a length that can vary from one week to three weeks. The energy prediction and the real measurement will be then compared, and, if the real measurement significantly differs from the predicted one, an alarm may be issued to let users know about the unusual situation. For the available period, from November 2020 to May 2021 (roughly seven months), data were organized in the following way hereinafter: from the beginning until 3 March 2021, samples were dedicated to training (60%); from that date until 16 April 2021, to validation (20%); and, finally, from that date until nearly the end of May 2021, for testing (20%). All the models considered hereinafter have been developed in Python 3.7 using Keras 2.2.4 and Tensorflow 1.13.1.

**Figure 3:**
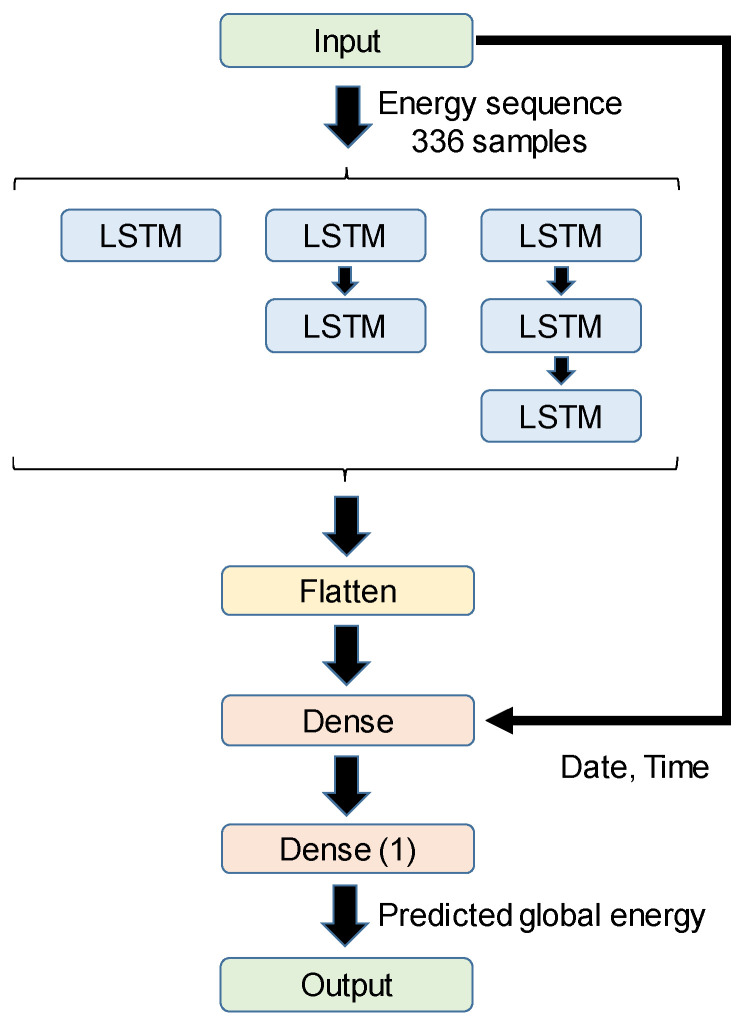
General scheme of the different LSTM topologies studied.

Firstly, the influence of the length in the input data was analysed for an LSTM network, since this defines the historic data used to predict the next consumption value. Three different lengths have been studied: one, two and three weeks before the next value. The network has a structure shown in Figure 3, that in the first iteration consists of an LSTM layer + LSTM layer + dense layer + dense layer, both with rectified linear units (ReLU). In all cases, we have not only provided the global consumption data of the house during the final considered days; we have also provided information about the current day of the week and the hour have been directly inserted at the input of the first dense layer after the LSTM layers. These are intended to provide the network with any additional information that may define the typical routines for each day of the week. Table 1 shows the results for the different lengths of the training window, for a learning rate of 10^−4^, a decay of 10^−6^, and a maximum number of epochs at 300, with early stopping and patience of 15 epochs. The optimization algorithm is Adam, and the validation loss function is the mean squared error. These training parameters have been chosen empirically, and are fixed throughout this work for the different tests presented hereinafter. Regarding the learning rate and the decay factor, remarkable improvement was not observed when varying them over several decades, both when making them smaller and larger. Concerning the number of epochs, the training sessions are either stopped prematurely with the early stopping mechanism, or they consume all epochs. In both cases, we have empirically chosen the number of epochs to be large enough to guarantee convergence.

It is possible to observe that the longer the training window is (three weeks), the lower the maximum absolute error (MAE) and the maximum squared error (MSE) are in the energy prediction. Nevertheless, at this point, there is a trade-off to be considered with regard to the complexity of the resulting neural network. Since the last goal is to integrate the prediction model in the smart meter itself, likely by means of an FPGA [40,41], in order to avoid a non-affordable increase in the complexity of the network, the training window of the consumption samples is fixed at two weeks hereinafter, since the resulting errors in the prediction are also suitable, while keeping a low computational complexity. 

A second aspect to study is the internal structure of the LSTM network. For that purpose, three options have been considered, by changing the number of LSTM layers from one to three, as is shown in Figure 3. In this case, Table 2 shows the prediction results, assuming a training window of two weeks, wherein MAE and MSE still stand for the maximum absolute error and the mean squared error, respectively. It is possible to observe that these small variations in the network’s structure do not actually have a relevant impact on the final performance at the expense of increasing the computational load, where the best balanced figures between error and complexity are obtained for the solution based on a single LSTM layer.

**Figure 4:**
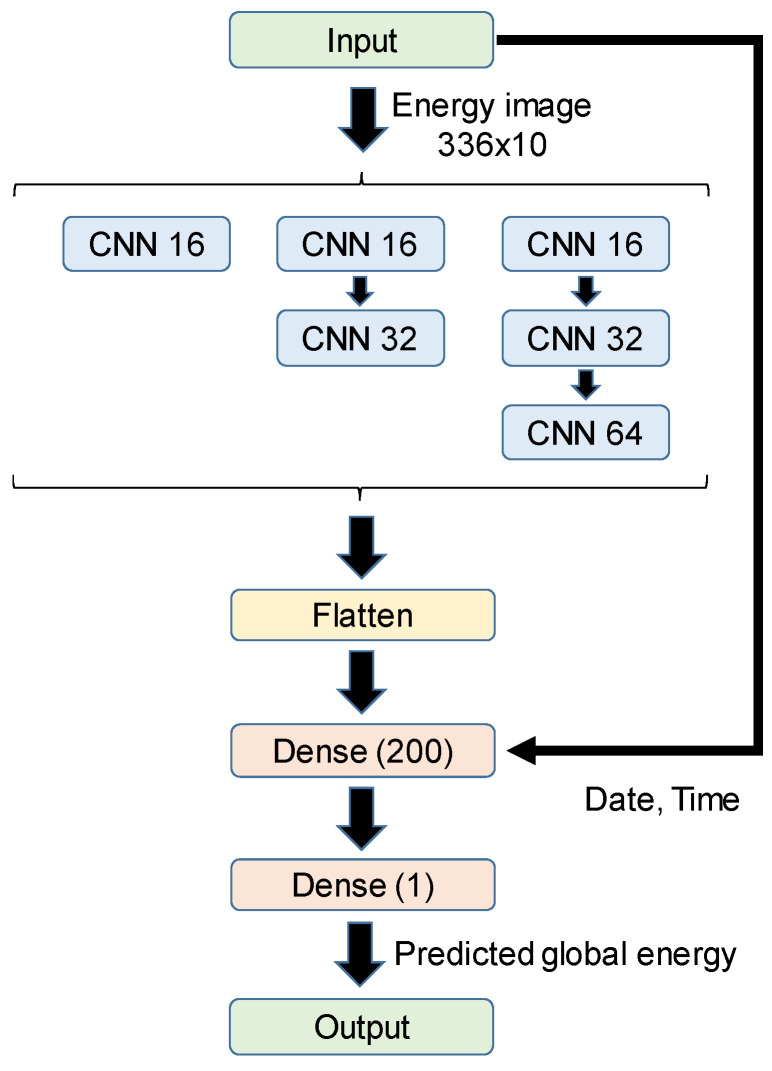
General scheme of the different CNN topologies studied.

A CNN structure has also been considered. As before, the input data are the energy samples for the last fourteen days every hour, together with the day of the week and the time. The energy samples are organized as an input image, with the size of 336 × 10, where 336 is the temporary depth (14 days × 24 h), and there are samples for ten different appliances. The internal CNN structure has been varied from one layer to three layers, increasing the filter from 16 to 32 and 64, and assuming a 3 × 3 kernel and a ReLU activation function. Figure 4 shows the general scheme of the CNN architecture. Table 3 shows the results for the different CNN topologies. On the other hand, assuming a structure with only one CNN layer (16 filters) and the same input, Table 4 shows the influence of kernel size on performance.

For comparison’s sake, similar tests have been carried out for a random forest (RF) approach, depending on the number of estimators involved in the topology. In this case, the input size still corresponds to a period of fourteen days, with a sample every hour, together with the day of the week and the time, meaning a total of 14 × 24 + 2 = 338 input samples. The performance is shown in Table 5. Similarly, with the same input, a solution based on a decision tree (DT) has been tested by varying the number of leaf nodes, as described in Table 6.

**Figure 5:**
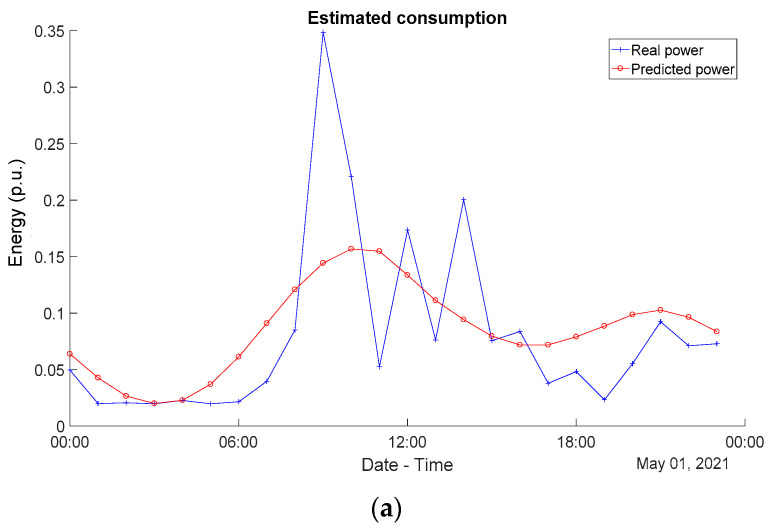

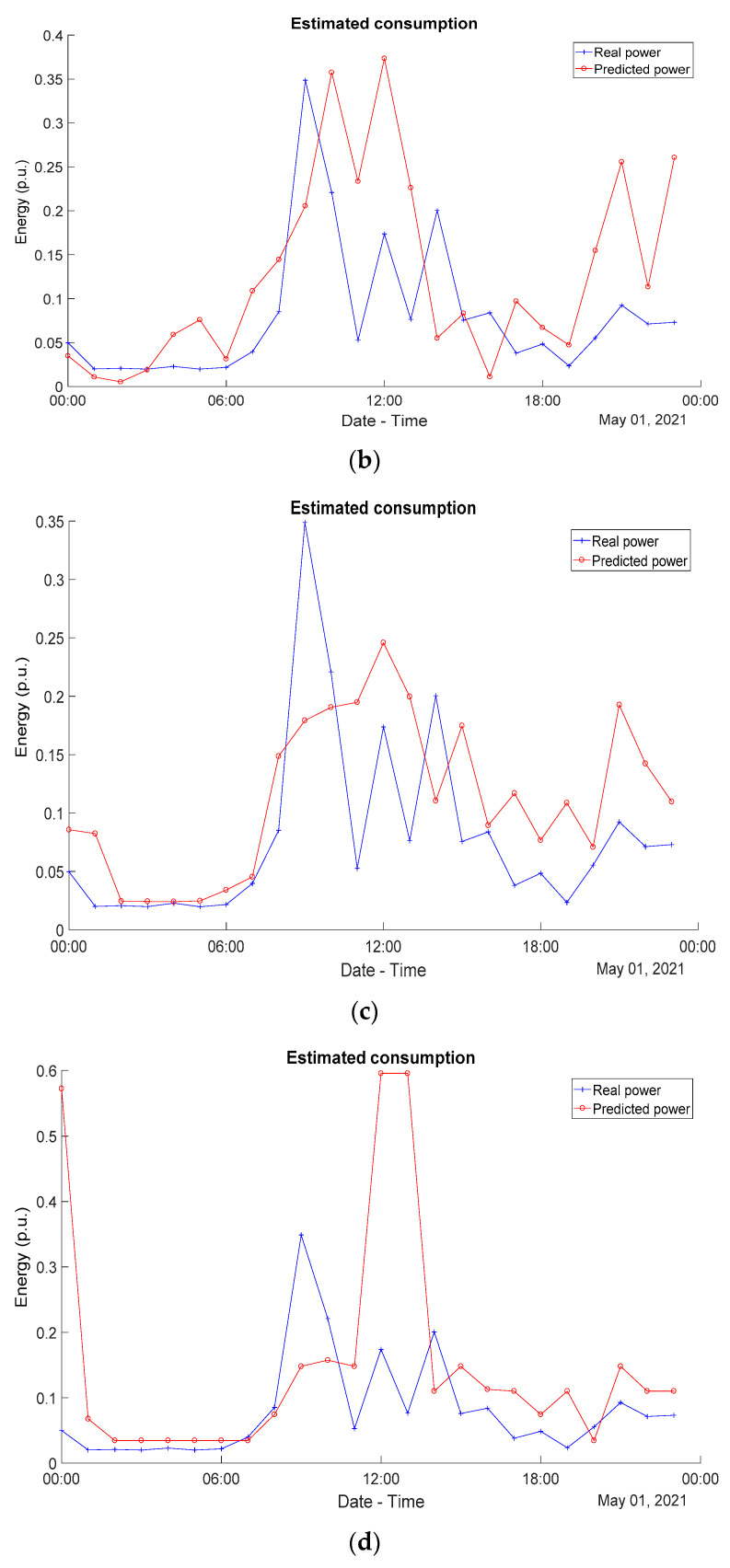
Real and estimated energy consumption on an hourly sampling basis (one sample per hour) for the four considered solutions: (**a**) LSTM; (**b**) CNN; (**c**) RF; and (**d**) DT. Note that the values have been normalized over a maximum of 4.5 kWh.

Taking into account all this information, Table 7 lists the best configuration for the four approaches studied in the test dataset: LSTM, CNN, RF, and DT. It is possible to observe that LSTM performs similarly to CNN, but with a higher computational load, whereas the random forest also achieves comparable results in terms of errors when predicting the global energy consumption. Furthermore, Figure 5 plots an example of the energy consumption predicted by the corresponding approach, as well as the real energy measured in the household for a single example day (the 1st of May). Note that energy samples have been normalized hereinafter at 4.5 kWh, since this is the maximum energy limitation existing in the household under analysis (actually, it is a common limitation in most houses in Spain). This normalization allows us to better fit the input and output data into the neural networks.

Despite the fact that the dataset involved is relatively small, the experimental tests presented hereinafter prove that the considered deep learning models do not suffer from severe overfitting. Specifically, the performance metrics in the test data are not significantly affected when increasing the number of training parameters in both the LSTM and CNN models. Table 1, Table 2, Table 3 and Table 4 illustrate that models with significant variation in the number of parameters do exhibit similar performance. Additionally, the results in Table 7 show that deep learning models are comparable in terms of MAE and RMSE to some classic machine learning models, such as RF, that are less prone to suffering from overfitting in this case.

## 4. Short-Term Alarm Generation

Based on the predicted and real energy consumptions, an alarm generator has been proposed for three different daily activities: breakfast, lunch, and sleeping. These activities were selected because they can be directly linked to the usage pattern of certain appliances, although the methods described hereinafter can be easily extended to other daily activities. This alarm generator is based on the following algorithm:

- The breakfast alarm *A_b_* is activated when the averaged predicted energy $\bar{p_{e}}$ provided by the model between 8 a.m. and 11 a.m. is higher than the averaged measured value or real one $\bar{p_{m}}$ for the same interval plus three times the standard deviation *σ_e_*[*n*] of the error between the predicted and the measured energies in that same interval (1). The limit of three times the standard deviation implies that the current difference between the predicted and the measured energies is significantly apart from the usual error from a Gaussian point of view. This means that breakfast activity did not take place as usual.

(1) $$A_{b}=\left\{\begin{array}{c} 1\;\;\;if\;\bar{p_{e}}>\bar{p_{m}}+3\cdot \sigma_{e}\\ 0\;\;\;if\;\bar{p_{e}}\leq \bar{p_{m}}+3\cdot \sigma_{e} \end{array}\right.$$

- The lunch alarm *A_l_* is activated in the range from 1 p.m. to 4 p.m. if the averaged predicted energy $\bar{p_{e}}$ by the forecasting model in that interval is greater than the averaged real one $\bar{p_{m}}$ plus three times the standard deviation of the prediction error for that interval, similar to (1), assuming then that the lunch was different from usual.
- The sleeping alarm *A_s_* is generated in the range from 12 p.m. to 6 a.m. when the averaged measured energy $\bar{p_{m}}$ is higher than the averaged energy consumption $\bar{p_{e}}$ predicted by the model plus the error standard deviation for that interval (2), indicating unusual activity during nighttime hours.

(2) $$A_{s}=\left\{\begin{array}{c} 1\;\;\;if\;\bar{p_{m}}>\bar{p_{e}}+\sigma_{e}\\ 0\;\;\;if\;\bar{p_{m}}\leq \bar{p_{e}}+\sigma_{e} \end{array}\right.$$

**Figure 6:**
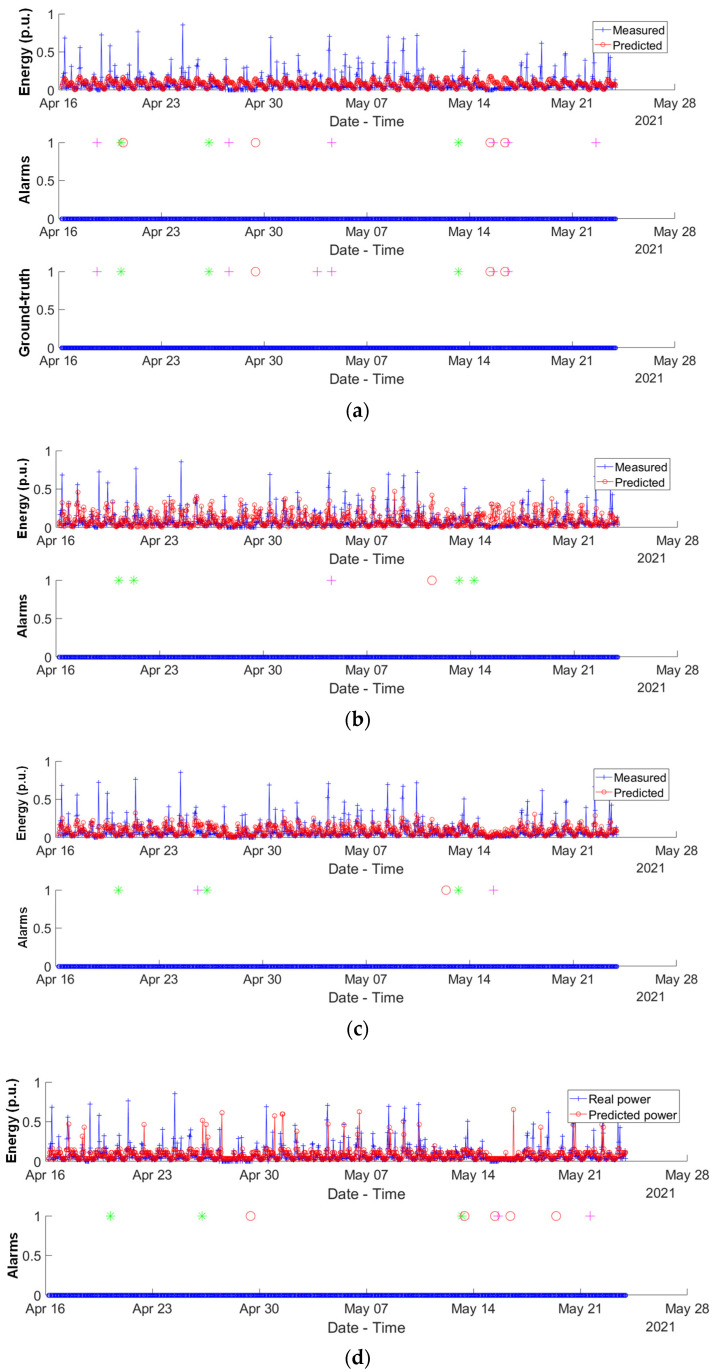
Activities’ alarm generation for the four considered solutions: (**a**) LSTM; (**b**) CNN; (**c**) RF; and (**d**) DT. The breakfast alarm *A_b_* is denoted by a red circle, the lunch alarm *A_l_* by a magenta cross, and the sleeping alarm *A_s_* by a green asterisk. Note that the ground truth is only included for the LSTM (**a**) because it is the same for the other topologies. The energy values are normalized over a maximum of 4.5 kWh.

**Figure 7:**
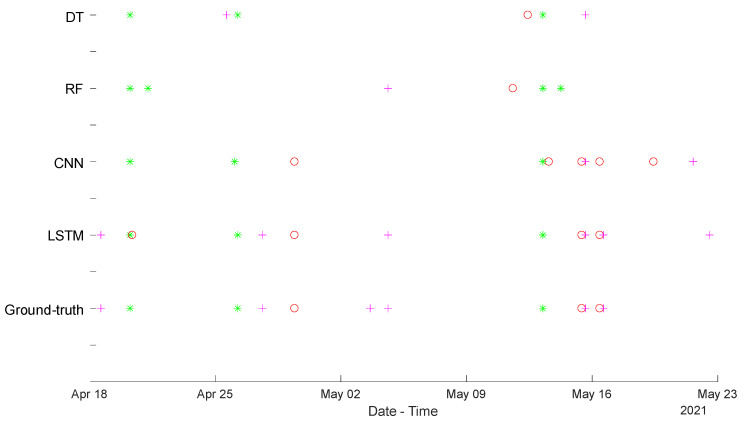
Summary of activities’ alarm generation for the four considered solutions: LSTM, CNN, RF and DT. The breakfast alarm *A_b_* is still denoted by a red circle, the lunch alarm *A_l_* by a magenta cross, and the sleeping alarm *A_s_* by a green asterisk. The energy values are normalized over a maximum of 4.5 kWh.

As was mentioned before, the proposals were trained with data from November 2020 to April 2021, and they were validated with available data from 16 April to 23 May. The alarm generator was tested for the three aforementioned daily activities, where the corresponding results are plotted in Figure 6. The breakfast alarm *A_b_* is denoted by a red circle, the lunch alarm *A_l_* by a magenta cross, and the sleeping alarm *A_s_* by a green asterisk. Furthermore, the plotted alarms can only be binary (1 for active and 0 for inactive). It is worth mentioning that the ground truth is provided in Figure 6a (it is the same for the other topologies); this ground truth was roughly and manually annotated by the dwellers in the household. For clarity’s sake, Figure 7 compiles the alarms generated by the four methods considered (LSTM, CNN, RF and DT), together with the corresponding ground truth, so they can be easily compared. It is possible to observe how two successive alarms for breakfast and lunch are generated on 16 May, corresponding to a weekend when the house remained empty.

Performance metrics have been obtained for the ADL alarm generation, according to the existing ground truth in Figure 6a. Table 8 provides these metrics, with which a limited analysis can be carried out due to the reduced number of alarm cases for the period under study (12 positive cases). Note that the accuracy, the precision, the recall, and the F1-score are calculated according to (3) and (4):

(3) $$Accuracy=\frac{TP+TN}{TP+FP+TN+FN}\;\;\;Precision=\frac{TP}{TP+FP}$$

(4) $$Recall=\frac{TP}{TP+FN}\;\;\;F1-score=2\cdot \frac{precision\cdot recall}{precision+recall}$$

where *TP* is the number of true positives; *TN* is the number of true negatives; *FP* is the number of false positives; and *FN* is the number of false negatives. Assuming that false negatives (*FN*) are the worst situation from a conservative point of view in this type of application, LSTM seems to provide the best performance, with the highest recall and F1-score.

## 5. Discussion

Monitoring the behaviour and routine patterns of persons living in a household in a non-intrusive way is a relevant field of research; it can provide interesting insights and information for different types of applications. The intelligent management of the energy demand is not only significant for companies, but also for end users that might benefit of better fees with the scheduled usage of certain electrical appliances. On the other hand, energy monitoring and forecasting are also an interesting tool in the domain of ambient intelligence for independent living. Particularly in Western countries, the ageing population is placing strain upon social and health services, and the search for novel remote health assistance systems and procedures is an open challenge. The possibility of installing sensors with a low level of intrusiveness in households to monitor and track the daily activities of tenants is a very attractive approach for specialists. In this context, smart meters may become a key device that may allow monitoring with almost negligible intrusiveness.

Data coming from smart meters can be processed and interpreted in many different ways. One option is that presented in this work, wherein a prediction model is developed to forecast future energy consumption based on the samples acquired in recent days or even weeks. Furthermore, the approach proposed here uses this prediction to launch a short-term alarm whenever the real energy consumption significantly diverges from the predicted series, since this implies variation in the usage pattern of electrical appliances. In this way, it is worth noting that the coarse-grain tracking of the daily activities in a house is feasible, and can be implemented using only a single-point sensor such as a smart meter.

Nevertheless, there are still some aspects and issues that are worth researching in the coming years. The first is the definition of intelligent systems that may be capable of monitoring activities in the long term, thus allowing the detection of possible variations in the behaviour of tenants. These variations are sometimes key for health specialists as an indication of a change in the health status of the patient. In this context, the type of activities and routines that can be inferred by smart meters are constrained to only those tasks involving the use of an electrical appliance, and many times only the most energy consuming appliances are clearly identified by NILM techniques. Recent studies have proven that higher sampling frequencies (in the range of kHz) and advanced signal processing, together with machine learning, may be able to identify loads with lower energy consumption, such as laptops, chargers, and so on. The identification of these devices would imply the recognition of more daily activities, thus enhancing behaviour monitoring.

Similarly, in more advanced scenarios, it is interesting to foresee how smart meters can be integrated in a more complex IoT (Internet of Things) sensor network, where other sensors, such as positioning, temperature, or smart devices (typically smartphones or smartwatches), can complement each other to obtain overall monitoring of the daily activity of a person. The main challenge here is how we may merge the different information coming from sensors in an efficient way, while keeping a low level of intrusiveness in the house under analysis. 

Another important aspect that we have not fully explored here and that deserves further investigation is the interpretability and explainability of AI backbones. Indeed, the LSTM network, which obtains the best results in alarm detection in our datasets, is a very complex black-box model whose interaction with the input signals cannot be easily seen or explained by a human. Classic machine learning methods, such as DT or RF, are highly interpretable and explainable, allowing us to rank the most important features that contribute to predictions and to evaluate biases in our datasets. However, their generalization and regression power is limited. On the contrary, the considered deep learning models are powerful, but not easily interpretable; thus, specific methods are required for analysing deep learning models and model agnostic approaches. This is an active topic of research that should be studied in future works, since explainable AI techniques might improve the training of models, by refining the importance of the features in the input signal.

## 6. Conclusions

This work presents the generation of activity alarms based on electrical consumption data from a commercial smart meter. These data are used to train an intelligent system capable of predicting the energy consumption of a household in the next one-hour interval. By comparing this prediction with the actual consumed energy, it is possible to identify the unusual completion of a certain daily activity, and, consequently, to launch a corresponding alarm, while maintaining negligible intrusiveness; this is because cameras, microphones and extra sensors are not required. Four different approaches have been studied for the design of the intelligent system: a recurrent network, a convolutional network, a random forest, and a decision tree. For experimental validation, a smart meter was installed in a household with four tenants, wherein data have been acquired and stored for a period of seven months. The resulting metrics indicate better performance in the case of the recurrent network, mainly due to the fact that it is more precise when detecting positive cases, thus reducing the number of false negatives and achieving a higher recall. In particular, the LSTM network proposed here has achieved a recall of 0.83 and an F1-score of 0.80, whereas the other models provide humbler figures below 0.58 and 0.63, respectively.

## Figures and Tables

**Table 1:** Influence of the length of the training window on the energy prediction based on an LSTM network.

No. of Weeks	1	2	3
No. of inputs	170	338	506
Structure: LSTM + LSTM + dense + dense	168/168/672/1	336/336/1344/1	504/504/2016/1
Trainable parameters	19,309,925	153,096,389	515,158,565
Batch size	70	68	65
MAE	0.0598	0.0587	0.0590
MSE	0.0105	0.0107	0.0102

**Table 2:** Analysis of the LSTM structure for a training input window of two weeks.

LSTM Structure	Structure Size	Trainable Parameters	MAE	MSE
LSTM + dense + dense	336/1344/1	152,191,877	0.0601	0.0106
LSTM + dense + dense	336/512/1	58,259,077	0.0589	0.0106
LSTM + LSTM + dense + dense	336/336/1344/1	153,096,389	0.0587	0.0107
LSTM + LSTM + dense + dense	336/168/512/1	29,697,061	0.0616	0.0107
LSTM + LSTM + LSTM + dense + dense	336/168/84/512/1	15,331,381	0.0611	0.0108

**Table 3:** Analysis of the CNN structure for a training input window of two weeks.

CNN Structure	Structure Size	Trainable Parameters	MAE	MSE
CNN + dense + dense	16/200/1	9,620,165	0.0645	0.0116
CNN + CNN + dense + dense	16/64/200/1	14,879,205	0.0728	0.1340
CNN + CNN + CNN + dense + dense	16/64/64/200/1	21,144,101	0.0716	0.0129

**Table 4:** Influence of kernel size on the CNN layer.

Kernel Size	Trainable Parameters	MAE	MSE
3 × 3	9,620,165	0.0645	0.0116
5 × 5	7,438,021	0.0664	0.0117
7 × 7	5,281,605	0.0627	0.0130
9 × 9	3,150,917	0.0657	0.0113
11 × 11	1,045,957	0.0597	0.0107

**Table 5:** Performance in the global energy prediction using a random forest.

Number of Estimators	MAE	MSE
400	0.0598	0.0098
200	0.0588	0.0097
100	0.0586	0.0097
50	0.0589	0.0099

**Table 6:** Performance in the global energy prediction using a decision tree.

Number of Leaf Nodes	MAE	MSE
50,000	0.0777	0.0211
10,000	0.0777	0.0211
5000	0.0777	0.0211
500	0.0759	0.0211
50	0.0640	0.0143

**Table 7:** Comparison of the four approaches considered (LSTM, CNN, RF, and DT) using the test dataset.

Topology	Features	MAE	MSE
LSTM	LSTM (336) + dense (512) + dense (1)	0.0589	0.0106
CNN	CNN (16, 11 × 11) + dense (200) + dense (1)	0.0705	0.0128
RF	200 estimators	0.0588	0.0097
DT	50 node leafs	0.0640	0.0143

**Table 8:** Performance metrics in the proposed ADL alarm generation.

	LSTM	CNN	RF	DT
Negative cases (NC)	895	895	895	895
Positive cases (PN)	12	12	12	12
True positive (tp)	10	3	4	7
True negative (tn)	892	892	893	892
False positive (fp)	2	3	2	3
False negative (fn)	3	9	8	5
Accuracy	0.99	0.99	0.99	0.99
Precision	0.77	0.75	0.67	0.70
Recall	0.83	0.25	0.33	0.58
F1-score	0.80	0.38	0.44	0.63

## Data Availability

Research data are unavailable at this moment due to privacy and ethical restrictions.
